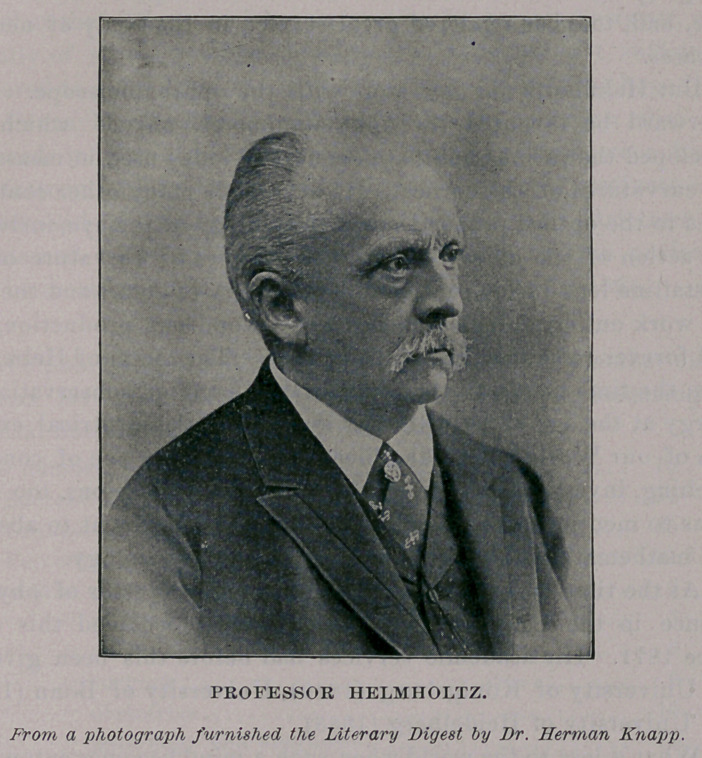# Professor Helmholtz

**Published:** 1894-11

**Authors:** 


### PROFESSOR HELMHOLTZ.

Baron Herman Ludwig Ferdinand von Helmholtz, better known in the scientific world as Professor Helmholtz, died in Berlin, September 8, 1894, aged seventy-three years. This event not only threw Germany into mourning, but throughout the scientific world, wherever science is known, there was sympathetic sadness. The cause of his death was a paralytic stroke, which

PROFESSOR HELMHOLTZ.

*From a photograph furnished the Literary Digest by Dr. Herman Knapp.*

occurred September 5th, consecutive to an initial stroke which he received early in July.

Professor Helmholtz was born at Potsdam, August 31, 1821, (his father being German and his mother English,) where he received his preliminary education and became a medical student, but took his final degree at the University of Berlin. After a brief service in the army in 1848 he was appointed professor of anatomy in the Academy of Fine Arts, and at the age of twenty-seven years he became professor of physiology in the University of Königsburg. It was during this period of his life that he turned his attention to physiological optics, and in 1851, at the age of thirty, he published a little treatise, entited *Beschreibung eines Augen-Spiegels zur Untersuchung der Netzhaut im Lebenden Auge*, in which he described the ophthalmoscope and the principles of its use. Could all else that Helmholtz ever did have been wiped out, this alone would have immortalized him. This instrument marked an epoch in ophthalmology, and has been the means of bringing about an immense advance in this department of medicine, and, too, has rendered great service in the study of nervous diseases.

But Helmholtz did not stop with the ophthalmoscope. Soon afterward he invented the ophthalmometer, out of which has developed the various ophthalmometers of today used in measuring the curvatures of the cornea. He also made many other contributions to the optical properties and physiology of the eye, including the action of the ciliary muscle, the changes of curvature of the crystalline lens during such action, the theory of colors and the like. His work on physiological optics was a wondrous production, and will forever remain a classic of our time. The career of Helmholtz from the time he wrote that remarkable essay on conservation of energy at the age of 26, till after his visit to the electrical exposition of our World's Fair at Chicago in 1893, was one of constant teaching, investigating and writing. His contributions, too numerous to mention here, were one continuous enrichment to abstract and mathematical science, to physics and to physiology.

At the time of his death, Helmholtz was professor of physical science in the University of Berlin, having occupied this chair since 1871. His academic services had before this been given to the University of Königsburg (1849), University of Bonn (1855), and University of Heidelberg (1865).

What a loss to the world when such a mind becomes entombed! It is no wonder that Germany is thrown into mourning for one of her greatest sons, and that the whole scientific world mourns with her, for the two hemispheres claimed this great man, and this sense of loss is not limited by territorial boundaries.

His funeral took place on September 12th, at Charlottenburg, and was attended by representatives of many universities and scientific societies, by Dr. Lucanias on behalf of the Emperor, and by Major-General Von Pfuhlstein on behalf of the Empress Frederick.

## Figures and Tables

**Figure f1:**